# Moulage safety practice around the world: a framework for best practice

**DOI:** 10.1186/s41077-026-00438-7

**Published:** 2026-04-14

**Authors:** Jessica Stokes-Parish, Daniel Bauer, Cecile Fourie, Maritza Harvey, Samantha McCormack, Jyotsna Rimal, David Shablak, Naomi Shiner, Janice Sinoski

**Affiliations:** 1 Faculty of Health Sciences and Medicine, Bond University, 14 University Drive, Robina, Queensland QLD 4226 Australia; 2 Institute for Medical Education, Faculty of Medicine, University of Bern, Bern, Switzerland; 3 University of the Free State, Bloemfontein, South Africa; 4 Clinical Skills Development Service, Metro North Hospital and Health Service, Herston, Queensland Australia; 5 College of Health, Medicine, and Life Sciences, Brunel University of London, Uxbridge, United Kingdom; 6 School of Medicine and Dentistry, Griffith University, Queensland, Australia; 7 United States Air Force-School of Aerospace Medicine, Wright-Patterson AFB, Ohio, United States of America; 8 Faculty of Medicine and Health Science, Keele University, Keele, United Kingdom; 9 School of Medicine and Health Sciences, University of Lynchburg, Lynchburg, Virginia United States of America

## Abstract

**Background:**

Moulage is ubiquitous with simulated participant (SP) practice. Moulage presents a physical and psychological risk to SPs, due to the application of active products, ingredients, and the creation of highly realistic wounds, which could evoke emotional distress or overidentification.

**Methods:**

The Moulage international Virtual Community of Practice convened in 2023 to commence standards of best practice in relation to moulage use with SPs. Using consensus methodology, the 9 authors catalogued their usual practice, reviewed relevant literature and standards to iteratively develop a conceptual framework and checklist.

**Results:**

As a result, a conceptual framework and checklist were developed for safe moulage practice for pre-simulation, during-simulation, and post-simulation phases. The framework highlights key roles and corporate governance to achieve risk mitigation regardless of the simulation setting. To augment the framework, the authors developed the Moulage Application Safety Checklist, a 17-item tool for moulage in use.

**Conclusion:**

The conceptual framework and checklist provide an evidence-informed tool for considering moulage safety with SPs. Safe use of moulage should not be an afterthought.

**Supplementary Information:**

The online version contains supplementary material available at 10.1186/s41077-026-00438-7.

## Introduction

Despite its ubiquity in simulation practice, the safety of moulage has not been explicitly considered. This work establishes a consensus-driven framework for safety when considering moulage.

Moulage, or the use of special effects makeup, has long been utilised in healthcare simulations due to its role in participant engagement, preparedness for trauma, and influence on participant satisfaction [1–4]. In this context, “moulage” encompasses all methods used to represent pathologies during simulation, such as makeup, transfer tattoos, or other special effects techniques [1]. Various substances (e.g., colour pigments, sealers, adhesives) applied in these processes have the potential to induce allergic reactions on SPs (individuals well trained to portray patients, clients, or family members) or other participants’ skin [5]. For example, latex is a known allergen frequently used in moulage techniques [6]. This paper reviews existing literature, current practice in moulage safety and sets out a framework for simulation practitioners to utilise when applying moulage in their setting.

### Existing literature

In May 2024, we (JS, JSP) searched the literature using a combination of keywords to identify scholarly sources on adverse events or safety practice associated with moulage use on SPs. Search terms included combinations of keywords such as moulage, allergic reactions, adverse reactions, psychological impact of moulage, moulage safety for standardized/simulated patients/participants, and moulage hazards. Three Boolean operators (AND, OR, NOT) were used to refine the search, and reference lists of key articles were also manually searched to identify additional relevant studies. The identified articles were screened by title, abstract, and then full-text to ensure inclusion criteria were met. Studies were included if they mentioned allergic or adverse effects in moulage use, were published in peer-reviewed journals, and were available in English. A search of five databases, including CINAHL, PubMed, Google Scholar, Embase, and ProQuest, yielded no relevant results. Empirical data on allergic reactions associated with moulage use could not be identified.

With no empirical data available, we explored the grey literature to inform practice. This included professional associations, companies that offer moulage training, and local legislation. Below is a narrative description of resources outside of traditional empirical evidence. Most sources refer to the application and removal of moulage and stage makeup without reference to the potential for physical or psychological adverse reactions to moulage [7, 8]. Stokes-Parish & Roiter (2022) offer strategic points to minimize infection during the application of moulage and provide images of common skin conditions linked to infections and allergies caused by moulage use [9]. Several grey literature references [7, 8, 10] mention the potential for allergic reactions, such as to latex, but an estimate of individual risk to SPs could not be found. Moulage is referenced in popular educational resources, such as HealthSimulation.org [11]. However, there is little to no guidance on safe moulage practice, or the risks associated with moulage use. Some companies reference safety in their training resources, for example, TraumaSim identifies the following learning objective – “how to safely plan moulage for a healthcare simulation educational or training experiences, including examples of safety considerations.” [12] However, these resources are not open-access or broadly accessible to the simulation user. Following reports of adverse events (such as inadvertently administering simulation fluids to real patients) [13], professional associations have developed standards of best practice to reduce risk and improve practice. For example, the International Nursing Association of Clinical Simulation and Learning (INACSL) developed Healthcare Simulation Standards of Best Practice (HSSBP) to ensure “all SBE experiences and associated activities are in an environment that complies with institutional, national, international, or other regulatory occupational safety practices” [14]. Specifically in the contexts of SPs, the Association of Standardized Patient Educators (ASPE) identified a safe work environment as the first of five domains for best practice, specifically identifying “anticipate and recognize potential occupational hazards, including threats to SP safety in the environment (e.g., allergenic substances, exposure to sharps, air quality, live defibrillators) [15]. The Association for Simulation Practice in Healthcare (ASPiH) standards describe the attributes required to deliver effective simulation-based education although safety guidelines for simulation are not specified [16].

Outside of the simulation literature, occupational safety legislation provides some guidance for practice. For example, in Australia, each state provides workplace safety standards related to cosmetics services, including recommendations for infection mitigation and personal hygiene through the Public Health (Infection Control for Personal Appearance Services) Act 2003 [17]. Other countries such as the United States of America provide guidance through the United States Food and Drug Administration or other, University-based guidance [18, 19]. In addition, peak bodies of the entertainment industry set safety guidelines for hazard identification and risk management [20].

Given the nature of SP work includes individuals of all ages and health backgrounds, such as children, older people, and those with preexisting skin conditions, we feel that this is a missed opportunity to take proactive action.

While this work focuses on SP safety, we should also consider the potential risks to all being exposed to moulage throughout the simulation process, including simulation faculty and participants.

While physical safety is most associated with moulage when considering risks, we must also consider the potential psychological effects of moulage. Recent research has highlighted the potential emotional impacts of using highly authentic moulage. Moulage might triggers sensory elements like sight, smell, touch, and hearing, and elicits strong emotions [21]. For example, in Fourie et al. (2023) respondents reported intense emotions after seeing and dealing with a patient with burn wounds [21]. Even the colour may affect human emotions and behaviour [22, 23]. For example, red could stimulate negative emotions with a reluctance to act [24, 25]. While the physical portrayal of moulage may have some influence, moulage may also prove an emotive trigger in scenarios such as melanomas, bruises in domestic violence scenarios, or burn injury simulations. This highlights the broader implications of simulations and their ability to evoke emotion [26]. Some research identifies using moulage to prepare participants for trauma patients. For example, Shiner and Howard used a simulated encounter with a patient with facial burns to prepare radiography students for clinical practice [27]. Students reported they felt more prepared to undertake their role in the imaging of complex care patients. Shiner further developed this work examining the role of moulage in emotional preparedness [4]. Shiner applied moulage to present a compound fracture on an SP, allowing students opportunities to engage with the patient and consider their emotions [28, 29]. Significant statistical differences were found in reducing negative emotions and increasing emotional preparedness following the simulation, indicating moulage has a role in reducing the risk of burnout in students [30]. Earlier literature highlights that portraying emotionally challenging scenarios can impact SPs negatively [31, 32]. According to a scoping review done by Lackie et al. 2023, Psychological or emotional safety is mostly achieved when skilled debriefers, debrief students after simulation activities according to established standards and models [33–41].

In this practice guideline, we report on experiences across an expert group of academic moulage specialists. This project convened key members of a virtual community of practice (vCOP) with the aim of developing principles for the safe use of moulage on SPs that would cover all aspects and be used in settings that are not well-resourced (such as lower-income countries and not-for-profit or volunteer organisations).

### Conceptual framework and safety checklist

For background, the moulage vCOP was formed in 2021 as an opportunity to bring together practitioners, researchers and simulation technicians globally to connect the moulage community and build collaborations for research and best practice guidelines. vCOPs are well documented as a useful method to collaborate in a social context to discuss common ideas, challenges, and deepen knowledge in a specific area [42–44]. The virtual community of practice is made up of moulage practitioners and researchers from all over the world, including Australia, Brazil, England, India, Nepal, South Africa, Switzerland, and the United States of America.

## Methods

**Fig. 1:**
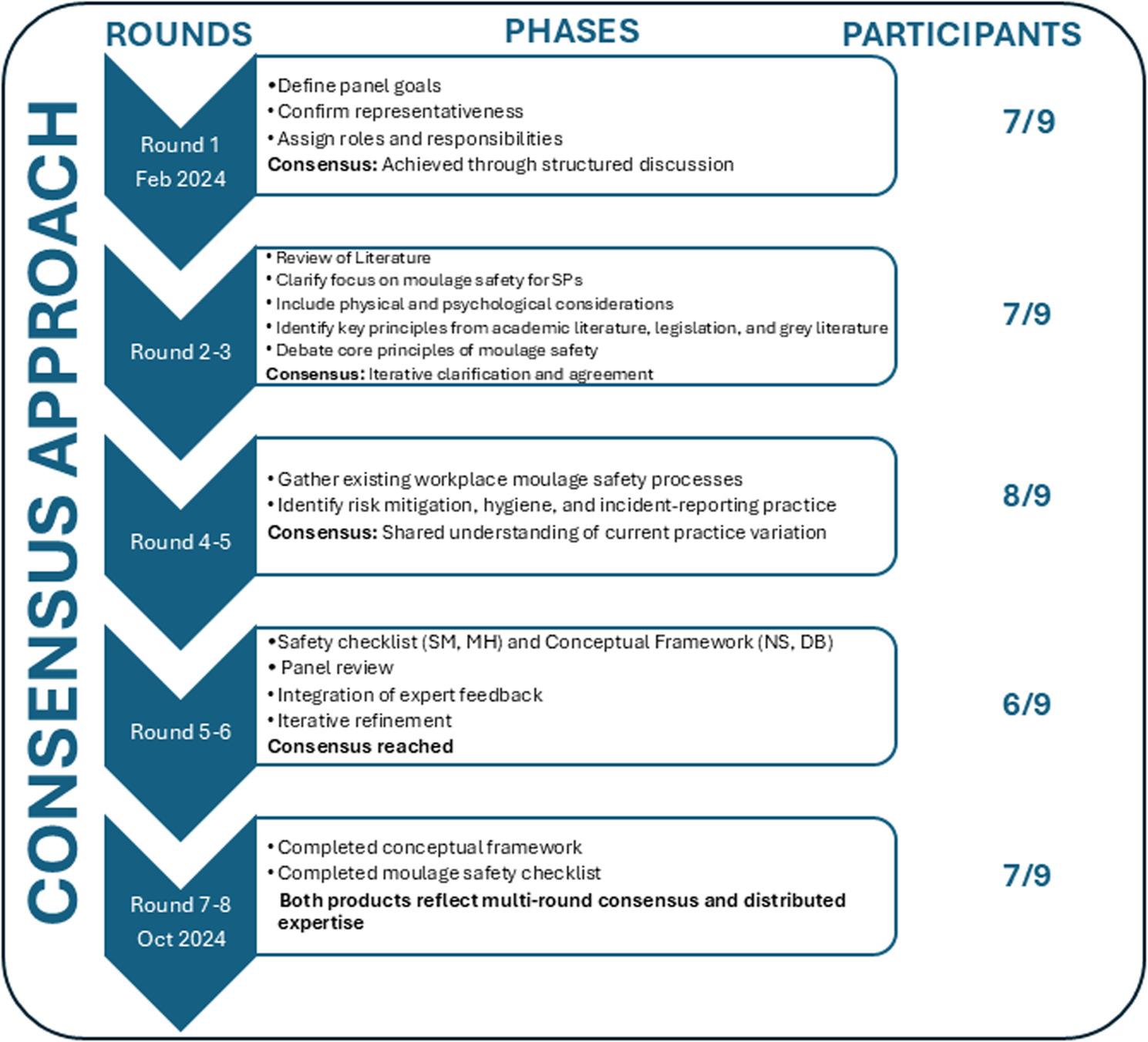
Consensus Approach

We utilised expert consensus methodology over iterative rounds informed by principles of virtual community of practice. Following the 2023 annual moulage virtual community of practice open meeting, members of the group identified gaps related to safety in moulage practice. 9 members of the virtual community of practice (vCOP) formed an expert panel to address this gap. A consensus approach was used, with panel members from Australia, Nepal, South Africa, Switzerland, the United States of America, and the United Kingdom. Members practiced moulage in simulation centres (*n* = 1), tertiary education (*n* = 6), and hospitals (*n* = 2). The panel comprised of individuals with academic and practical expertise covering burns, trauma, dermatology, general medicine and paediatric scenarios. They work in a variety of settings, including undergraduate and postgraduate tertiary sectors, a wide range of professions (including nursing, midwifery, medicine, radiography, and armed forces training). In addition, the specific, practical expertise included special effects makeup, hand-casting moulage, 3D printing and more. Our participants have a wide range of perspectives and experiences with moulage and SPs, which informed the discussion. No explicit research paradigm was established for the discussions. Over 8 meetings between February 2024 and October 2024, the panel developed the conceptual framework and safety checklist in five phases: establish the goal of the panel, refining the task, reviewing the literature, collated panel policies and procedures, and tool development (Figure 1 – Consensus Approach).

### Theoretical underpinnings

The term safety checklist is used to describe a tool used to aid safety in an organisation [45]. They are well-established in healthcare and other industries, such as military or airline management, and have been shown to improve quality of care and reduce human error. This approach was used to guide the practical development of the tool. Experts used their own experiences in moulage safety to contribute to the discussion.

### Establish the goal

We established the goal of the panel, explored the representativeness of the panel members, and established roles and responsibilities within the team.

### Refine the task

In this phase, we clarified the focus of moulage safety for moulage wearers (SPs) and that we would include both physical and psychological considerations. We identified the steps to take to identify literature and how we would determine inclusions and agreed that we would reach consensus iteratively.

### Review the literature

In rounds 2–3, panel experts identified and reviewed key principles from the literature, legislation, grey literature and debated the core principles of moulage safety. Experts iteratively discussed concepts until consensus on key aspects were reached. The literature search was conducted to inform framework development and did not constitute a systematic or scoping review.

### Collate policies and procedures Idea generation

Panel experts identified current moulage safety practice across their place of work. Members listed current processes related to moulage safety, including risk mitigation, hygiene, and incident reporting.

### Tool development and refinement

2 panelists (DB, NS) created the conceptual framework, and presented it in meetings 4 and 5 for panel review. 2 panelists (SM, MH) created the checklist and presented it in meetings 5 and 6 for panel review. The conceptual framework and checklist were adapted iteratively throughout each round, considering and integrating expert panelist feedback. Disagreements were managed by group discourse, with JSP acting as the primary mediator. To reduce the risk of gatekeeping or hierarchy, the principles of virtual communities of practice were upheld through identifying goals early on, offering multiple rounds of consultation, and allowing all to offer feedback throughout the rounds. Where panellists were unable to attend, they were provided an opportunity out of session to provide feedback.

## Results

### Current practice

**Fig. 2:**
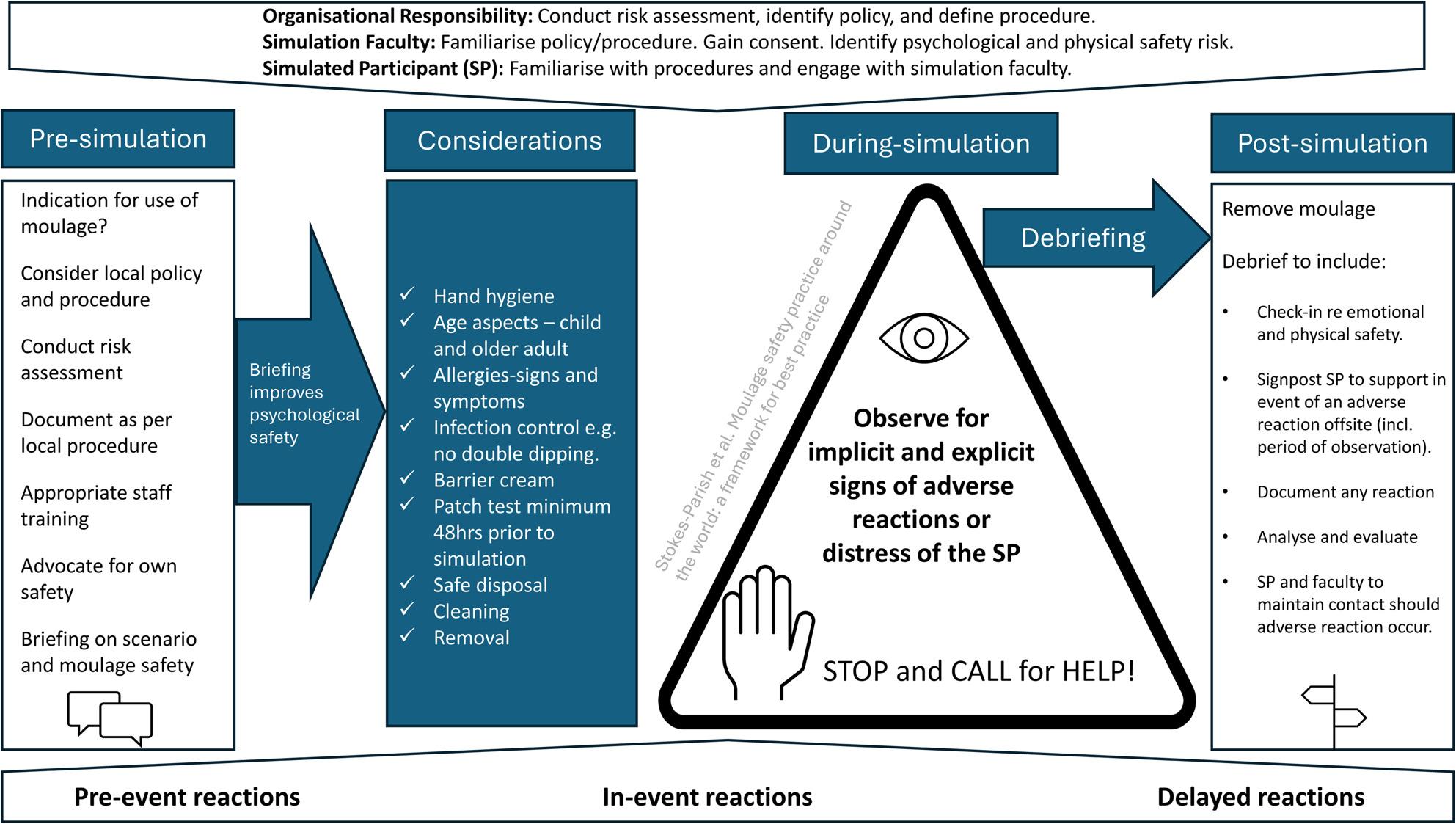
Conceptual Framework Moulage Safety

Safety practices associated with moulage use vary across locations. Of the authors listed, 7 of the 9 had formal moulage safety procedures in place. The authors work in higher education, hospitals, simulation centres, and the defence sector simulations. The safety practices implemented in these settings varied from extensive procedures to ad hoc, as needed. For example, in one large facility, a comprehensive pre-event checklist is completed 48 h prior to each simulation for each individual SP. This has a heavy administrative load, however, it allows tracking of any changes since the last application. In another facility, SPs are requested to advise of any allergies at the time of moulage application, and any incidents are reported via organisational workplace health and safety procedures. Considering the potential risk, the emerging evidence surrounding moulage use, and the expansion of best practice in simulation, it is time to identify principles of best practice for moulage. We produced a conceptual framework (Fig. 2 - Conceptual Framework Moulage Safety) and checklist for moulage safety in simulation. The conceptual framework is designed to be broad enough for universal use across a variety of contexts and settings. The conceptual framework draws from literature, expert practice and international standards of best practice to inform moulage safe practice. The framework broadly considers moulage in pre-simulation, during-simulation and post-simulation phases. Using an umbrella approach, the conceptual framework identifies key roles for the organisation, simulation faculty and simulated participant and the need for a strategic approach to achieve optimal outcomes.

### Implementing the framework – suggestions for practice

The conceptual framework is intended to be operationalised within the context of the user’s organisational structures, governance pathways, and simulation practices. The use of the framework does not negate the requirements of broader principles of clinical governance and risk management but supports the need for these principles to be applied to the use of moulage. Existing frameworks in occupational safety emphasises the proactive hazard identification, mitigation and clarity of roles to minimise potential harm. Generalists in health and workplace safety roles support the use of appropriate, practical, evidence-informed tools to identify workplace hazards without requiring extensive specialist training. The conceptual diagram and checklist form practical reminders and action points for this to occur [46]. Its purpose is to provide a systematic guide for decision‑making and professional judgement when employing moulage in simulated environments. The simplicity of the conceptual framework embraces human factor principles by reducing the cognitive load of the user and enhancing team communication checkpoints improving safe practice. Integrating these broader principles ensures that the use of moulage is not an isolated activity but one that is embedded within the organisations safety, accountability and continuous quality improvement strategies.

To utilise the framework effectively, users must first adopt a macro‑level perspective of their own role, responsibilities, and scope of practice. This reflective positioning enables practitioners to consider how their individual actions interface with organisational policies, risk management processes, and broader safety requirements.

At an organisational level, the framework encourages users to interrogate existing structures. For example, an initial question might be: *What formal risk assessments exist for the use of moulage in my organisation or setting?* In instances where no bespoke risk assessment is available, the framework directs users to review overarching health and safety policies such as infection prevention guidance, dermatological risk management, and psychological safety procedures and use these to inform the development of a standard operating procedure suited to their setting. This process ensures alignment between simulation practice and institutional regulatory expectations.

For simulation faculty, the framework emphasises the foundational requirement to be fully conversant with organisational policies and procedures. Compliance at this stage ensures appropriate protection for staff, learners, and simulated participants (SPs), and establishes a consistent baseline for safe practice.

The framework is then applied sequentially across the simulation event. In the *pre‑simulation phase*, users engage with the left-hand component of the framework, which prompts consideration of the necessity and appropriateness of moulage, review of relevant policies, completion of risk assessments, and documentation in accordance with local protocols. This stage also incorporates faculty preparedness, SP self‑advocacy, and pre‑briefing processes that strengthen psychological safety.

The *considerations* column functions as a structured checklist guiding users through key safety domains, including allergy assessment, infection control principles, hygiene requirements, the use of barrier products, patch testing procedures, disposal protocols, and appropriate removal methods. This section of the framework acts as an operational safeguard, ensuring critical factors have been addressed prior to moulage application.

During the *during‑simulation phase*, the framework centres on active monitoring of the SP. The triangular component of the diagram highlights the need for continuous observation for both implicit and explicit indicators of distress, discomfort, or adverse reactions. It foregrounds the importance of maintaining communication with SPs throughout the scenario and provides a clear directive for escalation (“STOP and CALL for HELP”) should any safety concerns arise. This embeds dynamic risk assessment as an integral part of the simulation process.

Following the event, the *post‑simulation phase* guides users through the structured removal of moulage, followed by comprehensive debriefing that includes emotional and physical check‑ins. It also establishes the expectation that any reactions or incidents should be documented, analysed, and evaluated. This cycle supports organisational learning and contributes to the refinement of policy, procedure, and practice in relation to moulage safety. The framework thus promotes a feedback loop in which SP and faculty reflections inform future operational improvements.

This framework is designed to be used in conjunction with the practitioner-focused tool (Supplemental File 1), The Moulage Application Safety Checklist (MASC). The MASC guides individuals to apply moulage to SPs from pre-simulation to post-simulation phase. The 17-item checklist considers general processes, pre-brief checklists, during simulation requirements, and after simulation debrief (including removal of moulage). This checklist also assigns roles to designated personnel to ensure safety standards are met.

In low-resource or volunteer-based settings, the framework and checklist could be used at the preparation and delivery stages to maximise safety. For example, if moulage product selection uses regular household pantry items, the framework accounts for the nuanced risks associated with their use. In practice, this might include avoiding high-risk allergen foods and ensuring immediate removal upon completion of the simulation to reduce skin sensitisation.

Overall, the conceptual framework and the MASC checklist provide a systematic, evidence‑informed approach to the safe and ethical use of moulage in simulation. By guiding practitioners through each stage, from pre‑simulation planning to post‑simulation evaluation, it enables consistent, transparent, and reflexive practice. The emphasis on communication, documentation, and organisational responsibility ensures that both immediate and longer‑term safety considerations are embedded within simulation-based education.

### Limitations

This work does not validate or assess the usability or reliability of the framework or checklist in practice, nor does it assess content validity. Future work should explore this in detail to assess the applicability across a number of setttings and might include further content validity studies or feasibility assessments in practice, including low-resource settings. For example, our experts geographic and institutional experience may introduce a bias in the inclusions of the framework. Further limitations include the absence of direct Simulated Participant involvement. The strengths of this work include the multi-nation and -discipline representation to develop a principles-based approach to a needed area of simulation.

## Conclusion

In conclusion, the current literature reflects a noticeable absence of empirical data collection regarding reported adverse reactions to moulage by simulation participants. The gap highlights the need to develop safe practice guidelines for moulage application and management in simulation. Forming an international, virtual community of practice we provided an opportunity to establish a collaborative platform for moulage specialists to discuss and reach a consensus on best practices in moulage safety, valuing each participant’s expertise and contribution. This international community of practice also serves as a key strength, enhancing credibility, broadening perspectives, and supporting the transferability of the work across diverse settings.

This framework developed through collaboration, offers a three-phased logical approach to managing safety in simulation, with a particular focus on moulage. The framework outlines the key responsibilities of the organisation, the simulation faculty, and the SPs in managing moulage use (and potential adverse events) during pre-brief, during-simulation, and debriefing.

The safety checklist facilitates clear communication to reduce omissions and errors and considers both the physical and psychological impact of moulage to promote safety for all simulation participants. However, there may be further potential risks associated with moulage use that we have not identified. We encourage the broader simulation community to consider investing in further exploration of moulage risks and safety and prioritize the simulation participants’ physical safety and psychological well-being.

## Supplementary Information

Supplementary Material 1.

## Data Availability

No datasets were generated or analysed during the current study.

## Glossary

Transfer: transferring a design (such as a prosthetic piece, tattoo, stencil, or template) onto the skin or another substrate to guide placement or fabrication.
Sealer: a protective coating applied to a surface to create a stable, uniform barrier.
Prosthetic adhesive: a medical‑grade or cosmetic‑grade bonding agent used to attach prosthetic appliances, medical devices, or special‑effects pieces securely to the skin.
Patch test: a small‑scale safety check performed by applying a tiny amount of a product (such as adhesive, sealer, or pigment) to a discreet area of skin.
SP: Simulated Participant
SPFX: Special Effects
SSH: Society for Simulation in Healthcare
MASC: Moulage Application Safety Checklist
vCOP: virtual community of practice
ASPiH: Association for Simulation Practice in Healthcare
ASPE: Association of SP Educators
INACSL: International Nursing Association of Clinical Simulation and Learning
HSSBP: Healthcare Simulation Standards of Best Practice
