# Supplementary material for: Moulage safety practice around the world: a framework for best practice
Source: Adv Simul (Lond). 2026 Apr 14;11:41. doi: 10.1186/s41077-026-00438-7 (PMC13188686; doi:10.1186/s41077-026-00438-7)
Supplement: Supplementary file 1 — Supplementary Material 1. [file 41077_2026_438_MOESM1_ESM.docx]

**Moulage Application Safety Checklist (MASC)**

This Moulage Application Safety Checklist is a guide for use when applying moulage to Simulated Participants (SPs). It ensures all necessary Health & Safety protocols are followed prior, during and after creating and applying moulage for simulation-based education.

| **Description** | **Checked/ completed by (date & initials)** | **Considerations** |
| --- | --- | --- |
| **General processes** | | |
| Complete Risk Assessment (RA) and store in line with Hospital / University policy |  | - List any known allergies (e.g., latex, adhesives).  - Document any medications that affect skin sensitivity  - Note any past adverse reactions to makeup or prosthetics  - Establish procedure for incidents and adverse events during and post-simulation |
| Complete SP education about the signs and symptoms of allergic reactions to moulage and what to do should a reaction occur |  | - Note organisational incident reporting process |
| Complete risk assessment related to products hazardous to health |  | - Store in line with your organisational process and policy |
| Before moulage is applied check SP* survey is completed - If they have allergy, risk management – refer to RA ** |  | - Obtain consent from SP detailing the moulage procedures according to organisational policy. |
| **Pre-brief checklist** | | |
| Set up moulage workstation |  | - Clean and controlled area for application.  - Check that the area is free from environmental allergens. |
| Use in date makeup *** |  | - Check expiration dates on all moulage products. |
| Wash and dry your hands |  | Follow policies and process for your organisation regarding sustainability |
| Consider psychological effect |  | - Explain process of application  - Pre-brief SP about realism and potential emotional impacts |
| Apply barrier cream |  | - Assess skin for skin conditions that may prevent moulage application prior to applying barrier creams to protect skin. |
| Individual makeup application using principles of “no touch” -, decanted, no double dipping, no using fingers |  | - Use individual makeup kits to avoid cross-contamination  - Use disposable applicators where possible |
| Clean and sanitise all tools after each SP application |  | - Ensure all tools and materials are sterilised and/or sanitised |
| **During simulation** | | |
| Report side effects after moulage is applied- refer to RA and incident reporting process |  | - Ensure emergency medical supplies are accessible |
| Check in on SP during simulation whilst moulage is on. |  | -Use pre-agree cues (verbal or non-verbal) for flagging concerns  -Assess presence of adverse reactions (physical or emotional) |
| **Post - simulation** | | |
| Remove moulage from SP |  |  |
| Report side effects after moulage is removed (if applicable) |  | - Refer to RA and incident reporting process |
| Psychological effect – Discuss their experience of wearing the moulage during the session |  | - Arrange for psychological support or debriefing if needed |
| Moulage expert to document all materials used according with organisational procedure |  | - Capture materials information (brand/lot/expiry), maintaining organisations material data safety sheet, noting compatibility (skin types/adhesives/removers), and setting storage/expiry policies. |

* SP – Simulated Participant– Adult or Child

**The agreement is required irrespective of whether one has been completed previously.

***Food products should be avoided due to potential allergic reaction

All cleansers used for cleaning equipment should be 70-90% alcohol based.
